# Effects of Age and Stand Density of Mother Trees on Early *Pinus thunbergii* Seedling Establishment in the Coastal Zone, China

**DOI:** 10.1155/2014/468036

**Published:** 2014-05-15

**Authors:** Peili Mao, Guangxuan Han, Guangmei Wang, Junbao Yu, Hongbo Shao

**Affiliations:** ^1^Key Laboratory of Coastal Zone Environmental Processes, Yantai Institute of Coastal Zone Research (YIC), Chinese Academy of Sciences (CAS), Yantai 264003, China; ^2^Institute of Life Sciences, Qingdao University of Science & Technology, Qingdao 266042, China

## Abstract

Effects of age and stand density of mother tree on seed germination, seedling biomass allocation, and seedling growth of *Pinus thunbergii* were studied. The results showed that age of mother tree did not have significant influences on seed germination, but it was significant on seedling biomass allocation and growth. Seedlings from the minimum and maximum age of mother tree had higher leaf mass ratio and lower root mass ratio than from the middle age of mother tree. Moreover, they also had higher relative height growth rate and slenderness, which were related to their biomass allocation. Stand density of mother tree mainly demonstrated significant effects on seed germination and seedling growth. Seed from higher stand density of mother tree did not decrease germination rate, but had higher mean germination time, indicating that it delayed germination process. Seedlings of higher stand density of mother tree showed higher relative height growth rate and slenderness. These traits of offspring from higher stand density of mother tree were similar to its mother, indicating significant environmental maternal effects. So, mother tree identity of maternal age and environments had important effects on natural regeneration of the coastal *P. thunbergii* forest.

## 1. Introduction

Plant regeneration is one of the problems in the ecological field in recent decades [1, 2]. The main regeneration stages of seed plants involve seed production, dispersal, and seedling establishment [1, 2]. Seed production is the first stage of plant regeneration. Seed quality and quantity are affected by maternal identity [3–7], such as maternal age [8, 9] and maternal environment [10, 11], which will influence the natural regeneration processes.

Mother tree age has significant effect on seed production. With the increase of age,* Cistus albidus* showed the highest fecundity at the middle age [9]. The seed production of* Picea mariana* [12] and* Pinus sylvestris* [13] increased steadily with mother tree age. Seed quality is affected significantly by tree age, too. Some tree species exhibited the highest seed germination rate in the middle age, such as* Sorbus torminalis* [14] and* Pinus echinata* [15].* Pinus pinea* increased seed vigour with the increase of mother tree age [10].* Pinus pinaster* increased seed germination time with increasing mother tree age [16]. However, Connor and Lanner [17] did not identify any relationship between tree age and pollen viability, seed weight, seed germination, and seedling biomass in* Pinus longaeva*. Müller et al. [9] also found that* C. albidus* did not decline in seed viability with increasing tree age. So, the relationship between tree age and seed production is ambiguous.

Maternal environmental effects enhance transgenerational plasticity in the maternal environment [18, 19], which have attracted considerable attentions in recent years [20–22]. As sessile organisms, the seed dispersal range of plant is often limited, with most seeds falling to the growth conditions of parents [23]. Parents' experiences from different abiotic stresses can preadapt offspring for functioning under the same stresses, which improves offspring fitness [21]. The maternal environment affects traits like seed traits [21, 24], germination [3, 18], seedling performance [5, 19, 21], and biotic stress [25, 26]. Environmental maternal effects can be inherited to the next generation and persist at least one generation [20, 21]. Moreover, Herman et al. [21] found that transgenerational environmental effects were cumulative over the course of two successive generations in* Polygonum persicaria*. The inherited adaptations did not change the DNA sequence but regulated gene expression [27]. Despite the substantial variation in transgenerational responses to stress observed, the molecular mechanism of environmental maternal effects remains poorly understood [22].

Coastal forests play a significant protective function in reducing natural disasters in the coastal zone [28, 29].* Pinus thunbergii* is one of the most important tree species in coastal forests of the Japanese islands and Shandong Province, China [30]. Because* P. thunbergii* forest near the sea is vulnerable to wind risk, few thinnings are carried out for their regeneration [29, 31, 32]. Due to few thinnings which lead to high stand density and low light intensity in the forest, the natural regeneration of the light demanding species* P. thunbergii* becomes very difficult [29–31]. Litter demonstrates negative effects on the regeneration [30, 31], which was similar to* P. pinea* natural regeneration [33]. Grass cover did not affect the survival and growth of the seedlings [30]. Grainger and Van Aarde [34, 35] thought the management of coastal forest restoration should be based on succession theory. However, the effect of seed production of* P. thunbergii* on its natural regeneration has not been researched systematically. So, we studied the influence of the mother tree age, the maternal environment, and their interaction, providing a quantification of the relative contribution of age and epigenetic effects on both the seed germination and the seedling growth.

## 2. Materials and Methods

### 2.1. Study Area and Species

The study was carried out at the coastal* P. thunbergii* forest around CAS Experimental Station of Integrated Coastal Environment in Muping, China (37°27′15′′N, 121°41′57′′E). It is a warm temperate continental monsoon climate in East Asia. The annual rainfall is 760 mm, and the mean annual temperature is 11.5°C. It is coastal sandy soil, whose soil organic matter content is less than 1%.* P. thunbergii* forest was planted in the 1950s and almost was pure. Understory vegetation was simple. Shrub species mainly consisted of* Amorpha fruticosa*,* Vitex trifolia *Linn.var.* simplicifolia *Cham.,* Lespedeza bicolor,* and* Rosa multiflora*. Grass species is made of* Carex rigescens*,* Corispermum stenolepis*,* Salsola ruthenica* lljin var.* ruthenica*,* Imperata cylindrica *var.* major*,* Portulaca oleracea*,* Calystegia soldanella*,* Ischaemum antephoroides*,* Artemisia capillaris* Thunb.,* Commelina communis*,* Atriplex sibirica,* and* Solanum nigrum*.

### 2.2. Cone and Seed Collection

Collection of the cones was carried out in late September of the year 2008. According to the census results of the community structure of the coastal* P. thunbergii* forest in 2007, we collected cones of different mother tree age classes (delegated by diameters at breast height) from a low (1600–1900 N/ha, D I) and a high (1600–1900 N/ha, D II) stand density, respectively (Table 1). We selected 10~15 individuals in each mother age class. More than 5 cones were randomly collected from each mother tree. Cones were air dried for several months until almost opened. Then, we selected filled seeds to use in the experiment.

### 2.3. Effect of Age and Stand Density of Mother Tree on Seed Germination

On 4 December 2009, 20 filled seeds of each pot were sowed in plastic pots of 0.2 m height and 0.22 cm diameter. The pots were filled with sand taken from the forest of cone collection and sieved to remove debris and seeds. Each treatment was determined by the age and stand density of mother tree (Table 1). Each treatment had five replicates. This experiment was conducted at the nurse garden of forestry bureau of Laishan District in Yantai, Shandong Provinces, China. The pots were laid randomly inside a low plastic tunnel. During the seed germination period, the minimal and maximum temperature in the tunnel varied from −6°C to 34°C. All pots were well watered to keep the soil near field capacity. After germinated seed was found on March 15, 2010, germination was assessed every day until April 28, 2010. Then, we evaluated germination rate (*G*_*r*_, %) and mean germination time (MGT, d). *G*_*r*_ was calculated as the ratio between the number of germinated seeds at a given time and the number of seeds sown. MGT was calculated as follows [36]: MGT = (*N*_1_ *T*_1_ + *N*_2_ *T*_2_ + ⋯+*N*_*x*_ *T*_*x*_)/total number of seeds germinated, where *N*_*i*_ = number of seeds germinating within consecutive intervals of time and *T*_*i*_ = the time between the beginning of the test and the end of the particular interval of measurement.

After the experiment end, five seedlings of each pot were removed from the pots and washed free of sand. Each seedling was divided into leaf, stem, and root, putted into a paper bag, and dried at 75 for 48 h. The masses of leaf, stem, and root were weighed by and electronic balance (0.1 mg accuracy). Individual seedling biomass (leaf + stem + root mass, g), leaf mass ratio (LMR, leaf mass/seedling biomass, g·g^−1^), stem mass ratio (SMR, stem mass/seedling biomass, g·g^−1^), and root mass ratio (RMR, root mass/seedling biomass, g·g^−1^) were accounted.

### 2.4. Effect of Age and Stand Density of Mother Tree on Seedling Growth

Three light levels were created by covering the low tunnel with layers of black nylon cloths that had little effect on radiation quality [37]. The light levels in different treatments were 50%, 30%, and 10% of the full light, respectively. Nine individuals of each pot leaved in the leaved seedlings of seed germination were transplanted into three pots on May 1, 2010 and each pot included three seedlings. Each pot was deposited randomly in different light levels, respectively. The growth experiment ended on December 30, 2010. During the experiment, seedling height and ground diameter were measured every month. We accounted relative height growth rate (RGR_*H*_, (ln*H*_2_ − 5 ln*H*_1_)/(*T*_2_ − *T*_1_), cm·g^−1^·d^−1^) and relative ground diameter growth rate (RGR_*D*_, (ln*D*_2_ − ln*D*_1_)/(*T*_2_ − *T*_1_), mm·g^−1^·d^−1^).* H* is seedling height (cm) and* D* is seedling ground diameter (cm). Subscripts refer to initial (1) or final (2) harvest. The slenderness was calculated according to Valladares et al. [38] by the following formula: slenderness = plant height/stem diameter.

### 2.5. Data Analysis

Seed germination responses were tested by using a two-way ANOVA, with age and stand density of mother tree as the source of variables. Three-way analysis of variance (ANOVA) was used to examine the main effects of light level, age, and stand density of mother tree and their interaction on seedling growth. Least-significant difference (LSD) multiple comparisons were conducted when there were significant differences. The statistically significant level was set at *P* < 0.05. All statistics were conducted with SPSS for Windows 13.0 (SPSS, Chicago, IL, USA).

## 3. Results

### 3.1. Seed Germination

**Figure 1:**
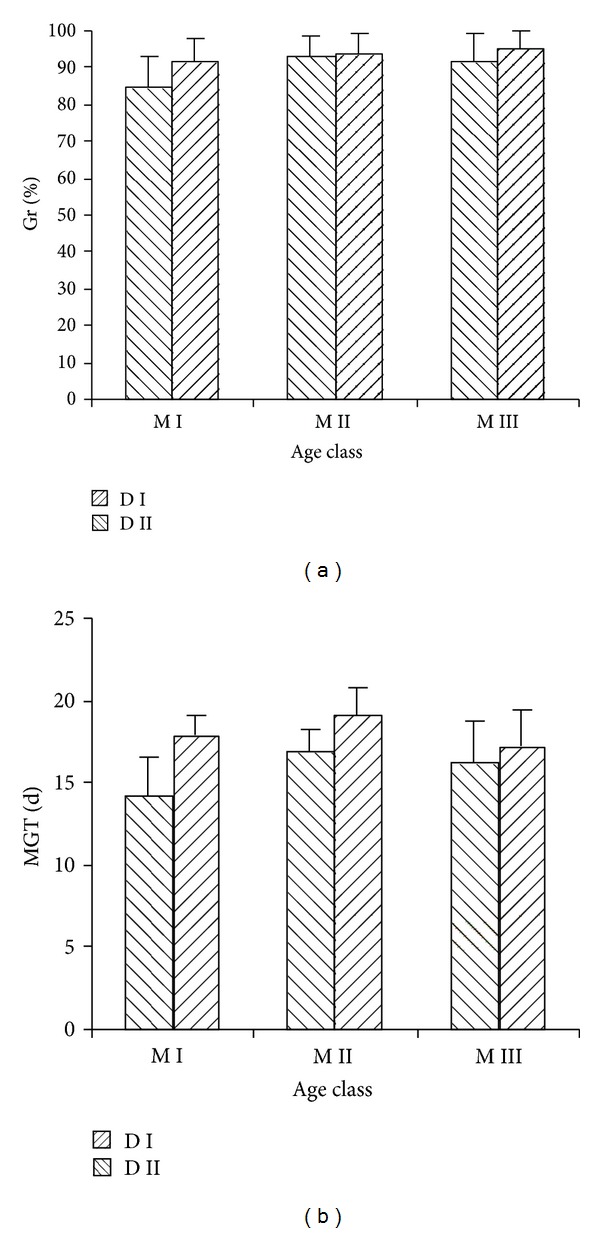
Effects of age and stand density of mother tree on seed germination.

Both age and stand density of mother tree did not show significant effects on *G*_*r*_ (Table 2; Figure 1). On MGT, age of mother tree had no significant effect, whereas stand density of mother tree exhibited significant effect. This result suggested that stand density of mother tree delayed germination time without changing seed quality.

### 3.2. Biomass Allocation

**Figure 2:**
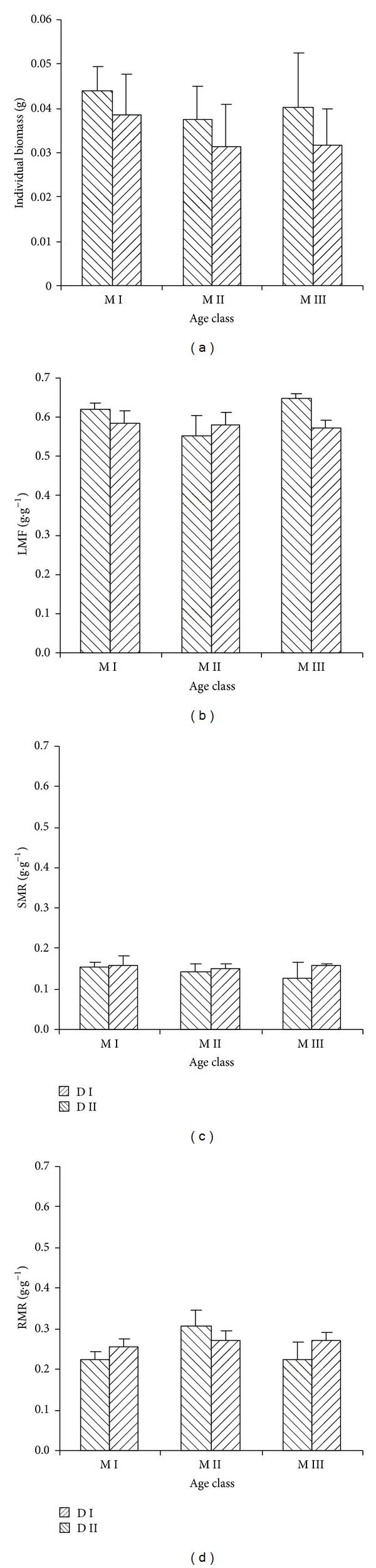
Effects of age and stand density of mother tree on seeding biomass allocation.

There were no significant differences on individual biomass both for age and for stand density of mother tree (Table 2, Figure 2). However, they showed different functions on biomass allocation. Age of mother tree showed significant effects on LMR and RMR but not on SMR. M I was similar to M III on LMR (*P* = 0.51) and RMR (*P* = 0.61). They were higher than M II on LMR (*P* < 0.05), but lower than M II on RMR (*P* < 0.01). Stand density of mother tree only exhibited significant effect on LMR. D I showed higher LMR than D II.

### 3.3. Seedling Growth

**Figure 3:**
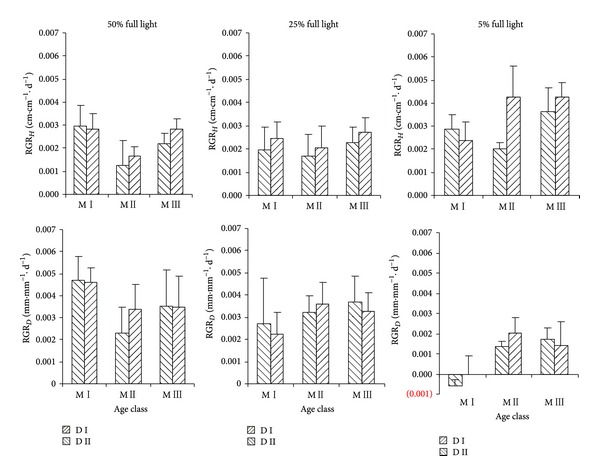
Effects of age and stand density of mother tree on seedling growth.

Light intensity had significant effects on RGR_*H*_ and RGR_*D*_ (Table 3, Figure 3). There were no significant differences on RGR_*H*_ (*P* = 0.93) and RGR_*D*_ (*P* = 0.19) between in 50% and 25% full light. However, they were lower on RGR_*H*_ (*P* < 0.01) and higher on RGR_*D*_ (*P* < 0.01) than in 5% full light. These results suggested that* P. thunbergii* was a light demanding species. Age of mother tree had significant effects on RGR_*H*_ not on RGR_*D*_. M I was similar to M II (*P* = 0.21) and M III (*P* = 0.11) on RGR_*H*_, while M II was lower than M III (*P* < 0.01). Stand density of mother tree also had significant effects on RGR_*H*_ not on RGR_*D*_. D I had higher RGR_*H*_ than D II in each light level.

**Figure 4:**
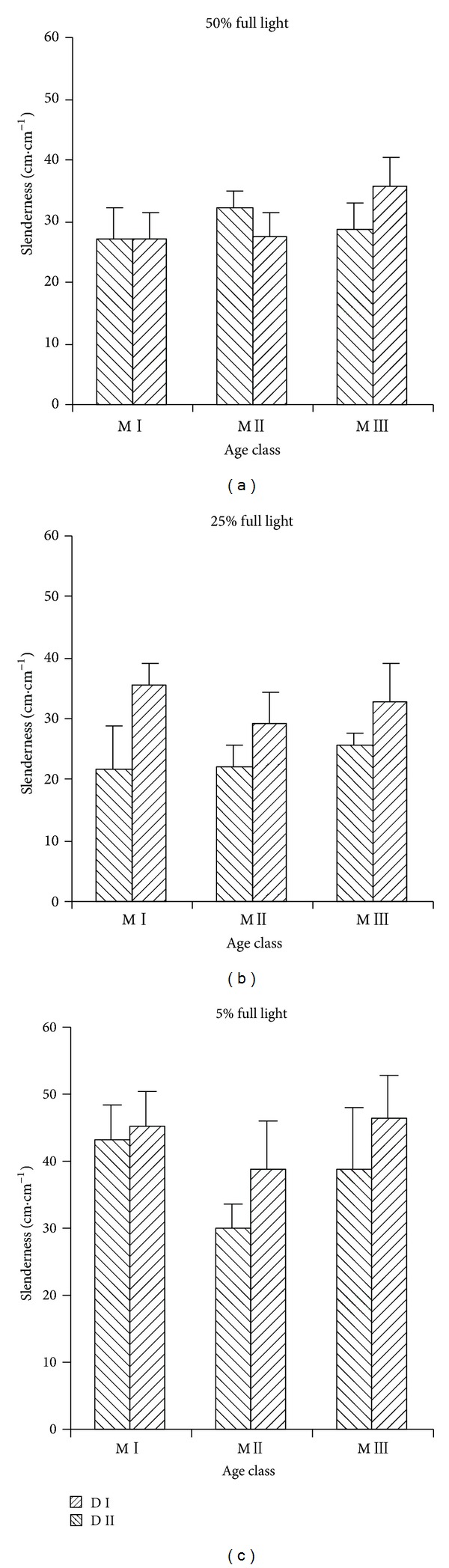
Effects of age and stand density of mother tree on seedling slenderness.

Light intensity had significant effects on slenderness (Table 3, Figure 4). There was no significant difference on slenderness between in 50% and 25% full light (*P* = 0.14), while they were lower than in 5% full light (*P* < 0.05). Age of mother tree showed significant effects on slenderness. M I was similar to M III on slenderness (*P* = 0.36), whereas they were higher than M II (*P* < 0.01). Stand density of mother tree also had significant effect on slenderness. D I showed higher slenderness than D II in all light levels.

## 4. Discussion

In this paper, we found that both tree age and maternal environment of* P. thunbergii* mother tree influenced the trait expression of its offspring. However, they showed differently functional manners in seed germination, seedling biomass allocation, and seedling growth, which suggested that mother tree character on their offspring showed complex impacts.

Seed germination is vital for the establishment of plant individual, especially in unfavorable environments [3, 22]. In this paper, we did not find significant effects of tree age on seed germination, which was in accord with Connor and Lanner [17] and Müller et al. [9]. Old* C. albidus* individuals kept high seed quality in harsh environmental conditions by decreasing their growth and photosynthetic biomass [9]. However,* S. torminalis *[14] and* P. echinata* [15] had the highest germination rate in the middle age of mother tree. Old mother tree of* P. pinea* showed higher germination rate than the younger [10]. Higher stand density of mother tree did not decrease germination rate but delayed germination time. In the* P. thunbergii* coastal forest, higher stand density had deeper litter and lower soil moisture [30, 31]. So, delay in the germination time of* P. thunbergii* in higher stand density was an adaptive manner in maternal environment. Similarly, seeds of* P. pinaster* from mother trees grown in the colder environment germinated 7.5 days later, which could help the offspring to escape from late frost damage [3].* Campanulastrum americanum* seed showed greater germination rate in their maternal light environment, which was an important adaptive mode to improve fitness for their offspring [18, 19].

Biomass allocation adjusting is an important adaptive mode for plant species to survive in different environmental conditions [39]. Both age and stand density of mother tree did not show significant differences in the early seedling growth. However, they demonstrated different effects on seedling biomass allocation, which would influence seedling establishment. Maternal age effect had significant influences on LMR and RMR not on SMR. M I and M III showed higher LMR than M II. High investment in leaf biomass is advantageous for plant species to survive in low light environment by increasing light interception ability [39] and keeping high photosynthetic capacity [40, 41]. So, M I and M III would have higher growth rate in understory. M II had higher RMR, which improved water acquisition [21, 42]. So, M II will favor to survive in high light environment. Stand density of mother tree only influenced leaf mass ratio. D I had higher leaf mass ratio than D II, which seemed D II did not have growth advantage in understory. We thought this pattern would change with different environment conditions in its later growth process.

Growth integrates the effects of different stresses on plant species vigour and carbon balance [43]. With the decrease of light intensity,* P. thunbergii* seedling increased RGR_*H*_ and decreased RGR_*D*_, which confirmed it was a light demanding species [44]. Rapid height growth was advantageous for plant species to shade surrounding shorter plants and reach canopy [45]. Both age and stand density of mother tree showed significant effects on RGR_*H*_, suggesting height growth had vital function for the regeneration of* P. thunbergii*. M II exhibited the lowest RGR_*H*_, which was related to its lower leaf mass ratio [46, 47]. D II had higher RGR_*H*_, especially in 25% and 5% full light, which was in accord with higher stand density of mother tree. Plant slenderness suggested its vigour [38]. Seedling under 5% full light had the highest slenderness, which indicated they were biomechanically weaker. Zhu et al. [30] found that all of the regenerated seedlings in the unthinned* P. thunbergii* stand were only 1-year-old. So, we thought low vigour of seedlings was an important reason for the failure of the establishment in understory. Higher slenderness for D II indicated that seedling was slimmer, which suggested significant environmental material effects.

## 5. Conclusion

Due to harsh physical environments in the coastal shore, natural regeneration for the coastal* P. thunbergii* forest is very difficult. Seed production is the first step of natural regenerationfor* P. thunbergii*. In this paper, we found that maternal identity had important role on their offspring' seed germination, seedling biomass allocation, and seedling growth. However, there were different functions between age and stand density of mother tree. The age of mother tree influenced seedling growth by altering their biomass allocation. The stand density of mother tree influenced seedling establishment by environmental maternal effects on seed germination and seedling growth. So, it is an important research to clarify more maternal environmental effects. Moreover, we should also consider maternal effects and physical environments and their interaction in order to elucidate natural regeneration mechanism of the coastal* P. thunbergii* forest.

## Figures and Tables

**Table 1:** Mother tree characteristics of *Pinus thunbergii* for collecting cones and seeds.

Age class	D I		D II	
	Diameter at breast height (cm)	Height of mother tree (m)	Diameter at breast height (cm)	Height of mother tree (m)
M I	9.55~12.74	6.1~7.3	6.37~9.55	5.6~6.4
M II	12.89~15.92	6.5~9.2	10.51~12.74	6.4~8.6
M III	16.56~19.11	6.8~9.8	12.89~15.92	6.3~8.6

**Table 2:** Two-way ANOVA analyses of effects of age and stand density of mother tree age and their interaction on seed germination and seedling biomass allocation for *Pinus thunbergii. *

	Age		Density		Age × Density	
	*F* value	*P* value	*F* value	*P* value	*F* value	*P* value
Germination rate (Gr)	0.49	*P* = 0.62	1.27	*P* = 0.27	0.44	*P* = 0.65
Mean germination time (MGT)	0.96	*P* = 0.40	9.45	*P* < 0.01	0.66	*P* = 0.53
Individual biomass	1.33	*P* = 0.28	3.93	*P* = 0.06	0.06	*P* = 0.95
Leaf mass ratio (LMR)	5.44	*P* < 0.05	6.89	*P* < 0.05	6.79	*P* < 0.01
Stem mass ratio (SMR)	1.06	*P* = 0.36	2.28	*P* = 0.15	0.96	*P* = 0.40
Root mass ratio (RMR)	6.38	*P* < 0.01	1.94	*P* = 0.18	4.35	*P* < 0.05

**Table 3:** Three-way ANOVA analyses of effects of light level, age, and stand density of mother tree age and their interactions on seedling growth for *Pinus thunbergii. *

Source	RGR_*H*_		RGR_*D*_		Slenderness	
	*F* value	*P* value	*F* value	*P* value	*F* value	*P* value
Light	8.60	*P* < 0.01	38.30	*P* < 0.01	41.20	*P* < 0.01
Age	4.69	*P* < 0.05	1.49	*P* = 0.23	5.72	*P* < 0.01
Density	4.98	*P* < 0.05	0.31	*P* = 0.58	19.53	*P* < 0.01
Light × age	2.24	*P* = 0.08	6.27	*P* < 0.01	3.14	*P* < 0.05
Light × density	0.47	*P* = 0.63	0.39	*P* = 0.68	4.36	*P* < 0.05
Age × density	1.73	*P* = 0.19	1.24	*P* = 0.30	0.63	*P* = 0.53
Light × age × density	1.30	*P* = 0.28	0.16	*P* = 0.96	2.01	*P* = 0.11
